# High volume online post-dilution hemodiafiltration: how relevant is it in chronic kidney disease?

**DOI:** 10.1590/2175-8239-JBN-2021-0172

**Published:** 2022-02-02

**Authors:** Manuel Carlos Martins Castro

**Affiliations:** 1 Universidade de São Paulo, Faculdade de Medicina, Hospital das Clínicas, Serviço de Nefrologia, São Paulo, SP, Brasil.

**Keywords:** Renal Dialysis, Hemodiafiltration, Kidney Failure, Chronic, Mortality, Diálise Renal, Hemodiafiltração, Falência Renal Crônica, Mortalidade

## Abstract

Online hemodiafiltration is potentially a superior mode of dialysis compared to conventional hemodialysis. However, prospective randomized controlled trials have failed to demonstrate such superiority. Post-hoc analyses of these trials have indicated that high volume post-dilution hemodiafiltration is associated with lower death rates than conventional dialysis. This study discusses whether the lower death rates ascribed to high volume hemodiafiltration are linked to convection volume or the time on dialysis needed to achieve high convection volumes.

## RESUMO

A hemodiafiltração on-line é uma modalidade de diálise com potencial de superioridade sobre a hemodiálise convencional. Entretanto, estudos prospectivos, randomizados e controlados falharam em demonstrar a superioridade da hemodiafiltração. Análises post hoc desses estudos sugerem que a hemodiafiltração pós-dilucional de alto volume apresenta taxa de mortalidade inferior à hemodiálise convencional. Neste estudo, discutimos se a menor taxa de mortalidade da hemodiafiltração de alto volume está associada ao volume de convecção ou ao tempo de diálise necessário para atingir um alto volume de convecção.

## INTRODUCTION

Despite the improvements made to dialysis equipment and supplies, death rates still remain elevated. In 1981, the National Cooperative Dialysis Study (NCDS) showed that increases in dialysis dose were associated with decreases in death rates^1^. In the NCDS, two variables were considered in order to alter the dose of dialysis: blood flow rate and treatment time. Statistical analysis showed that increases in blood flow rate and urea clearance were the factors more significantly associated with death rate decreases. Treatment time had a marginal, non-statistical effect^1^.

The reanalysis of the data of the NCDS performed by Gotch & Sargent^2^ and Keshaviah & Collins^3^ using urea Kt/V confirmed that higher dialysis doses were associated with lower treatment mortality. The bases for what would be called high efficiency hemodialysis (HD) had been established.

Based on these findings, efforts were made to establish the conditions needed to increase urea clearance by increasing blood flow rate, the surface area and ultrafiltration capability of dialysis filters, and dialysate flow rate. Attempts to extend time on dialysis were not made, since patients, physicians, and dialysis staff have resisted the idea, and a national study conducted in the United States failed to report lower death rates when time on dialysis was increased.

In this setting, the last four to five decades have seen the development and introduction in clinical practice of different approaches to hemodialysis aimed to decrease mortality. Large multicenter randomized controlled trials comparing different dialysis modes have been organized in the past 15 years, but their combined outcomes have not been analyzed. This study interprets and discusses previously published trials in an integrated, sequential manner.

## DISCUSSION

The MPO (Membrane Permeability Outcome) study looked into the impact of high flux membranes on the survival of incident dialysis patients^4^. The analysis of the survival curves did not show a significant difference between low and high flux membranes. However, patients with serum albumin levels below 4 g/dl in the high flux membrane group had significantly better survival than their counterparts in the low flux membrane group. In addition, secondary analysis revealed that high flux membranes improved the survival of patients with diabetes. Treatment time was not controlled for in the MPO study, and adjustments were made only to ensure a minimum single pool Kt/V (spKt/V) of 1.2.

The HEMO (Hemodialysis) Study evaluated the effects of dialysis dose and high flux membranes on patient mortality^5^. Although small differences were seen in the results observed for specific patient subgroups, the authors of the study concluded that neither the increases in dialysis dose nor the use of high flux membranes were associated with lower mortality. Dialysis time was not controlled for in this study. In the group of patients given the standard treatment dose (equilibrated Kt/V = 1.16±0.08), time on dialysis was 190±23 min, while in the high dose group (equilibrated Kt/V = 1.53±0.09) treatment time was 219±23 min. Although treatment took 30 minutes longer in the group prescribed high dose dialysis, the time difference between groups was not substantial enough to allow assertions about potential effects of treatment time on death rates. Actually, the HEMO study showed that in shorter dialysis sessions (between 3 and 3.5 hours) the increase in the equilibrated Kt/v from 1.16 to 1.53 did not yield significant benefit.

The DOPPS (Dialysis Outcomes and Practice Patterns Study) is a multicenter prospective observational study with more than 20 years of duration that included a wide array of patients from multiple dialysis units in more than 20 countries. The study went through several phases and enabled the assessment of different factors connected to dialysis approaches.

In regard to treatment time, the DOPPS showed that patients attending hemodialysis sessions at the care center three times a week had significantly lower mortality when dialysis sessions were longer^6^. Statistical analysis found that increasing dialysis time by 30 minutes decreased mortality by 7%^7^. The study also suggested that lower mortality might be associated with the decreased ultrafiltration rates used in longer dialysis sessions^7^.

In countries where hemodialysis sessions are longer, such as Australia and New Zealand (255±41 min), mortality is significantly lower than in the United States, where hemodialysis sessions are shorter (212±32 min)^6^. However, despite extensive adjustments and the use of an instrumental variable approach, the potential for residual confounding factors remains and results cannot establish a cause-effect relationship between dialysis time and improved clinical outcomes.

Observations of this type have certainly indicated a need for prospective randomized controlled trials to assess the importance of treatment time for mortality in dialysis.

The development of synthetic and semisynthetic dialysis membranes with elevated ultrafiltration coefficients, coupled with strict control over ultrafiltration rates during dialysis sessions and the development of equipment that allows the online production of large volumes of replacement fluids, led to the incorporation of dialysis approaches based on convective (hemofiltration) and convective-diffusive (hemodiafiltration) transport in clinical practice.

Isolated reports have suggested that patients treated with convection dialysis have longer survival. Three prospective studies were designed to assess this hypothesis.

The Dutch CONTRAST (CONvective TRAnsport STtudy) study compared patients on low flux hemodialysis against individuals on post-dilution hemodiafiltration (HDF)^8^. No differences were seen in all-cause mortality or cardiovascular death between groups. However, the reanalysis of study results showed that patients with a convection volume greater than 21.9 liters/session had significantly lower death rates (relative risk = 0.62; 95% CI, 0.41-0.83). Treatment time was not controlled for in the CONTRAST study. Treatment time was set at the start of the study and was increased only when the spKt/V dropped to below 1.2. Post-hoc analysis of the results showed that for convection volumes < 17.9 liters, between 17.9 and 21.8 liters, and > 21.8 liters, treatment times were 214±26 min, 229±21 min, and 235±16 min, respectively (p < 0.001)^9^.

A study performed in Turkey (Turkish OL-HDF Study) compared patients on high flux hemodialysis and individuals on post-dilution hemodiafiltration^10^. The composite result for all-cause mortality or non-fatal cardiovascular event was not different between groups. However, reanalysis of study results showed that patients with a replacement volume greater than 17.4 liters/session had significantly lower death rates (relative risk = 0.71; 95% CI, 0.07-0.71; p = 0.01). Treatment time was not controlled for in this study, but information extracted from the article allow the estimation that for replacement volumes of 15.9 liters, 17.2 liters, and 18.5 liters, dialysis times were 230 min, 236 min, and 242 min, respectively^10^.

The Catalan study ESHOL (Estudio de Supervivencia de Hemodiafiltración OnLine) compared patients predominantly on high flux hemodialysis (92%) and individuals on post-dilution hemodiafiltration^11^. During randomization, patients failing to reach a convection volume of 18 liters/session were excluded. In this study, the mean convection volume was 23.7 liters/session. Patients assigned to the HDF group had a 30% decrease in the risk of all-cause mortality (95% CI, 0.53-0.92; p=0.01) and a 33% decrease in the risk of cardiovascular death (95% CI, 0.44-1.02; p=0.06). Compared to hemodialysis, the relative risk of death was 0.60 (95% CI, 0.39-0.90) for convection volumes between 23.1 and 25.4 liters and 0.55 (95% CI, 0.34-0.84) for convection volumes greater than 25.4 liters/session. Treatment time was not controlled for in this study, but information extracted from the article allow the estimation that for convection volumes < 23.1 liters, between 23.1 liters, and 25.4 liters, and > 25.4 liters, treatment times were 231 min, 242 min, and 254 min, respectively^11^.

The FRENCHIE (French Convective versus Hemodialysis in Elderly) study conducted in France compared 381 elderly patients (age > 65 years) randomized 1:1 into high flux HD or online HDF^12^. Death rates at 13 and 24 months were 11.0% and 22.5% in the HD group and 8.9% and 18.9% in the HDF group. All-cause mortality and cardiovascular deaths were not different between the two groups (p = 0.43). Patients on HDF had their death rates compared between groups with convection volume < 20 liters (16.5±3.0 liters) and > 20 liters per session (25.5±6.1 liters), and no significant differences were found between groups. Mean dialysis session duration was 3.93 hours in the HD group and 3.94 hours in the HDF group. Convection volume was 2.0 liters in the HD group and 21.2 liters in the HDF group (p < 0.001). Therefore, when dialysis times were similar, differences in mortality between HD and HDF tended to disappear.

Peters et al. conducted a study taking individual patient data from the last four clinical trials cited above^13^. The analysis of the effects of HDF compared to HD indicated that HDF decreased the risk of mortality, with larger effects among patients offered larger convection volumes. Unfortunately, the authors did not look into the relationships between treatment time, convection volume, and death rate.

In a similar analysis involving the same four studies, Davenport et al. also found that greater convection volume was associated with longer survival in subjects on HDF. However, the authors found that for convection volumes of 18.0 (16.0-18.8), 21.0 (20.2-22.0) and 25.7 (24.4-27.4) liters (median-interquartile range), the mean dialysis times were 226±23, 234±14, and 240±18 minutes, respectively^14^.

Finally, the European arm of the DOPPS study carried out from 1998 to 2001 and published in 2006 revealed that patients on HDF with replacement volumes between 15.0 and 24.9 liters per session had a relative risk of death of 0.65 compared to the low flux hemodialysis group (p = 0.01)^15^. However, the authors did not present any information about the duration of dialysis sessions.

On the other hand, the analysis of the results of Phases 4 and 5 of the DOPPS conducted from 2009 to2015 and published in 2018 showed that the risk of death was 1.14 (95% CI, 1.00-1.29) for HDF versus HD, and 1.08 (95% CI, 0.92-1.28) for HDF with a replacement volume > 20 liters versus HD^16^. Similar results were found for cardiovascular and infection-related death. In this analysis, treatment times for HD and HDF with replacement volumes between 4 and 15 liters, between 15.1 and 20 liters, and > 20 liters were 238±26, 234±30, 240±26, and 249±31 minutes, respectively^16^. Therefore, when similar treatment times were considered, the results did not support the notion that HDF improves patient survival.

In summary, there is no consensus about the superiority of HDF over HD. Controlled randomized trials failed to show differences in death rates. However, some differences became apparent when patients were grouped according to convection volume. For replacement volumes > 20 liters or convection volumes > 22 liters per session, there is a significant reduction in mortality among subjects on HDF. From this finding stemmed the concept of high volume hemodiafiltration.

However, studies comparing between different dialysis modes using high flux membranes must be interpreted with caution. Due to the elevated membrane ultrafiltration coefficient characteristically seen in HD sessions with high flux membranes, reverse ultrafiltration is a constant concern and an element that yields unknown convection volumes. In a way, every dialysis session that uses high flux membranes is an HDF session^17,18^.

**Figure 1:**
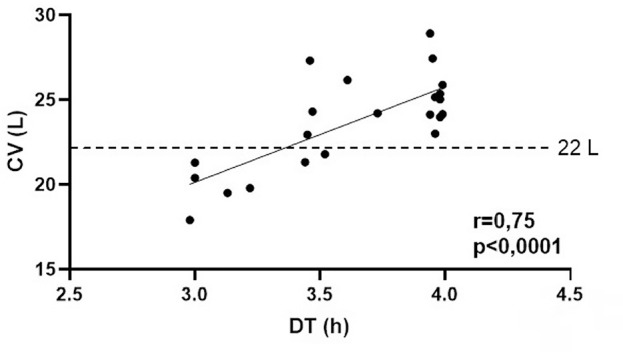
Correlation between dialysis time (Dt) and convection volume (CV) of patients on online post-dilution hemodiafiltration.

In high volume online post-dilution HDF the main factors determining convection volume are session duration, blood flow rate, filtration fraction, and hematocrit value^19^. Using yet unpublished results extracted from the database of a single-center prospective quality control study, we looked into the relationship between treatment time and convection volume of 22 patients on online post-dilution HDF. Our patients underwent 1449 HDF sessions (65.8±15.5 sessions per patient; M±SD) each lasting for 3.6±0.4 hours, with an online Kt/V of 1.53±0.28, and a convection volume of 23.6±2.8 liters per session. In nine (41%) patients, the mean convection volume ranged between 17.9 and 21.8 liters, and in 13 (59%) patients the mean convection volume ranged between 22.9 and 28.9 liters. We found a strong correlation between dialysis time and convection volume (r = 0.75; p < 0.0001) (Fig. 1).

**Figure 2:**
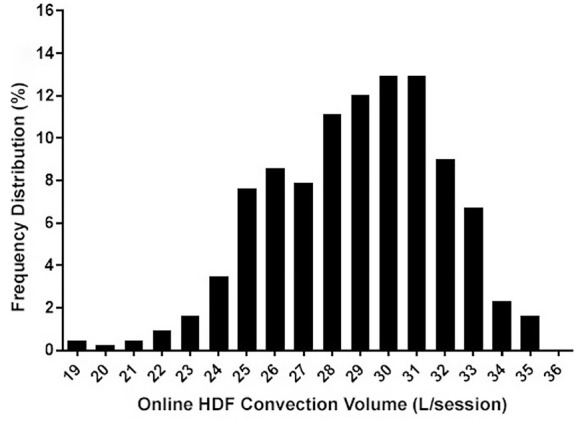
Pattern of convection volume distribution in four-hour online post-dilution hemodiafiltration sessions.

Marcelli et al. looked into variables that may be managed to reach a minimum replacement volume of 21 liters in HDF^20^. In this study, the authors set the minimum effective dialysis time at 4 hours and adjusted blood flow rate and filtration fraction to reach the target volume. In 79% of the HDF sessions a replacement volume ≥ 21 liters was achieved; this percentage was even higher when only fistulae or grafts were considered (86.9% and 83,8%, respectively). Using the database mentioned previously, we assessed the convection volumes of 432 HDF sessions with treatment time set at 4 hours and an effective blood flow rate ≥ 350mL/min. The median convection volume was 29.1 liters (Q1-Q3: 26.6-31.0; min-max: 19.0-35.4), and in only 2.1% of the HDF sessions convection volume was below 22 liters (Fig. 2).

These results clearly show that, in addition to setting dialysis time at four hours, one needs to control other variables to achieve a sufficient convection volume for HDF to be regarded as of high volume.

Rating high volume online HDF as a superior dialysis method is an idea that needs confirmation in randomized controlled trials designed to rigorously monitor potential confounding variables and duration of dialysis sessions in particular. This is particularly important since the relationships between convection volume and mortality were extracted from re-analyses and post-hoc analyses of large prospective studies.

The international prospective randomized controlled trial CONVINCE was designed with that idea in mind and intends to compare primary outcome all-cause mortality and secondary outcomes cause-specific mortality, cardiovascular events, all-cause hospitalization, infection-related hospitalization, quality of life, and therapy cost-effectiveness for patients prescribed high flux HD and high dose HDF. High dose HDF was defined as treatment with a convection volume ≥ 23±1 liters/session^21^.

The trial aims to enroll 1800 patients with chronic kidney disease randomized 1:1 to high flux HD and high dose HDF. Patients will be followed for 36 months (minimum of 24 months). The trial started in May 2019 and by March 2021 it had enrolled 1360 patients in eight countries.

More than 85% of the patients on HDF achieved a convection volume ≥ 23 liters/session^22^. To get to this volume, the protocol permits adjustments to three variables: treatment time (210, 240 or 270 minutes); blood flow rate (300, 350 or 400 ml/min); or filtration fraction (21-31%). The protocol did not prescribe a fixed treatment time. Depending on the patient, the target convection volume may be achieved with different treatment times.

Therefore, the comparison of outcomes of patients on high flux HD versus the outcomes of subjects on high dose HDF may be influenced by treatment time. Ideally, to avoid bias, treatment time should be fixed for the two treatment modes. The same might occur when HDF outcomes with different convection volume ranges are compared, since results might be more significantly affected by treatment time than convection volume.

Therefore, the CONVINCE trial might not definitively show that the decreases in mortality reported in some studies about high volume HDF stemmed from greater convection volume and not longer treatment time, which is needed to achieve greater convection volumes.

The recently published Australian study FINESSE (Filtration in the Neuropathy of End-Stage kidney disease Symptom Evolution) was designed to assess neuropathy progression in patients randomized to high flux hemodialysis (n = 61) or high volume hemodiafiltration (n = 63)^23^. The study median duration was 41 months. Weekly treatment time was the same in the two therapy modes (14.8±0.2 hours) (p = 0.79; M±SEM). The spKt/V was 1.6±0.04 in HD and 1.56±0.05 in HDF (p = 0.56; M±SEM) and convection volume in HDF was 24.7 (22.4-26.5) liters/session (median-interquartile range).

Thirty-two deaths occurred during the study, 15 in the high flux HD group and 16 in the high volume HDF group, indicating the absence of a difference in survival (hazard ratio 1.24 (0.61 - 2.51), log rank p = 0.55). This study presented limitations, but indicated that when treatment time was long and similar, there was no difference in mortality between subjects on high flux HD and patients on high volume HDF. These findings indicates that studies designed to compare survival rates in high flux HD and high volume HDF must consider controlling treatment time.

## CONCLUSION

The nephrology community has been encouraged to transfer patients from conventional hemodialysis programs with high flux and high efficiency membranes to high volume online hemodiafiltration protocols. However, this measure requires substantial financial investment, which may be a significant burden for developing nations. Even in developed countries, the transference of patients from high flux HD to HDF has been limited^24^.

Considering that many studies have found that mortality in dialysis is lower with longer treatment times^25^ and that in order to obtain high convection volumes treatment time needs to be extended, at the moment it is not possible to state that increases in survival in high volume HDF are associated with convection volume and not with increases in treatment time needed to achieve high convection volumes.
